# B.1.617.2 (Delta) Variant of SARS-CoV-2: features, transmission and potential strategies

**DOI:** 10.7150/ijbs.66881

**Published:** 2022-02-14

**Authors:** Yan Zhan, Hui Yin, Ji-Ye Yin

**Affiliations:** 1 Department of Clinical Pharmacology, Xiangya Hospital, Central South University, Changsha 410078, P. R. China; Institute of Clinical Pharmacology, Central South University; Hunan Key Laboratory of Pharmacogenetics, Changsha 410078, P. R. China.; 2 Engineering Research Center of Applied Technology of Pharmacogenomics, Ministry of Education, 110 Xiangya Road, Changsha 410078, P. R. China.; 3 National Clinical Research Center for Geriatric Disorders, 87 Xiangya Road, Changsha 410008, Hunan, P.R. China.; 4 Hunan Key Laboratory of Precise Diagnosis and Treatment of Gastrointestinal Tumor, Changsha 410078, P. R. China.

**Keywords:** COVID-19, B.1.617.2, Delta variants, SARS-CoV-2, transmission, vaccines, diagnosis, structural biology, immune escape

## Abstract

Coronavirus disease 2019 (COVID-19) caused by the severe acute respiratory syndrome coronavirus 2 (SARS-CoV-2) has become a pandemic. With the continuous evolution of the viral genome, SARS-CoV-2 has evolved many variants. B.1.617.2, also called Delta, is one of the most concerned variants. The Delta variant was first reported in India at the end of 2020 but has spread globally, by now, to 135 countries and is not stand still. Delta shared some mutations with other variants, and owned its special mutations on spike proteins, which may be responsible for its strong transmission and increasing virulence. Under these circumstances, a systematic summary of Delta is necessary. This review will focus on the Delta variant. We will describe all the characteristics of Delta (including biological features and clinical characteristics), analyze potential reasons for its strong transmission, and provide potential protective ways for combating Delta.

## Introduction

Severe acute respiratory syndrome coronavirus 2 (SARS-CoV-2), the causative pathogen of Coronavirus disease 2019 (COVID-19), emerged in late 2019 1. Because of the alarming levels of spread and severity, COVID-19 was characterized as a pandemic on March 11, 2020. Since the outbreak of the epidemic, a large number of lives have died in the process of fighting the virus. To date, the cumulative number of infection is nearly 212 million and the number of cumulative deaths is 4.4 million. COVID-19 has led to a serious public health crisis, and poses a huge threat to daily life.

The first whole genome sequence of SARS-CoV-2 was available within ten days after the occurrence of clustered cases 2. Several months later, three million genome sequences were submitted. A great quantity of mutations obtained by analyzing these sequences reveal the variability of SARS-CoV-2 genome. Actually, it was not surprising that SARS-CoV-2 has new variants, because RNA virus evolves and changes easily. The mutations we can detect were those that can survive and spread successfully 3. Since the first COVID-19 genome sequence was uploaded to the database, scientists began to monitor the evolution of SARS-CoV-2 in real time.

The emergence of B.1.1.7 has attracted attention 4, 5, because there is no previous evidence that variation will increase the adaptability of SARS-CoV-2 genome. In general, a new variant may increase circulation frequency or the change of clinical characteristics. B.1.1.7, also known as Alpha variant, was first reported in the United Kingdom. It exhibited a significant increase in transmission 6, 7 and was listed as variant of concern (VOC) by the World Health Organization (WHO) 7. Currently, it has distributed all over the world. Mutations in Alpha variant make it easier for the virus to bind to the surface receptor of target cells, which greatly enhances the infectivity. Studies have shown that the infectivity of viral variant is increased by about 50% when compared with the original virus. In addition to B.1.1.7, there are some other VOCs, including B.1.351 (Beta) 8, P.1 (Gamma) 9, B.1.617.2 (Delta) and B.1.1.529 (Omicron). The possibility of SARS-CoV-2 variants with distinct characteristics to evolve was increased due to the immense number of current cases. B.1.351 starts in South Africa, and P.1 in Brazil, the outstanding feature of these two viruses is that they have the ability to escape immunity 10. In other words, they may lead to decreased vaccine efficacy and ineffective drug treatment. Delta is also a VOC. Compared to the original virus, Delta has the following four features: (1) high viral load. In terms of viral load, Delta is 1,260 times that of last year's pandemic strain; (2) large exhaled virus concentration. It makes people more likely to be exposed to the virus; (3) highly contagious disease; (4) un-conspicuous symptoms. The two aspects mentioned later make Delta difficult to prevent. This review focused on the features and transmission of B.1.617.2 variant. The potential strategies against Delta will also be discussed.

## Features of Delta variant

Recently, WHO announced that viruses within the lineage B.1.617 have been characterized as VOC or variant of interest (VOI). B.1.617 contains three sub-lineages, which included B.1.617.1 (also known as Kappa), B.1.617.2, and B.1.617.3 11. B.1.617.2 lineages have been divided into VOCs, which may affect the transmission ability of the virus, sensitivity to vaccine and its pathogenicity to humans. B.1.617.1 and B.1.617.3 lineage were VOIs that are significant variants requiring further observation 12.

### Biological characteristics of Delta variant

In general, Delta virus is a variant of SARS-CoV-2, so their biological characteristics were similar to SARS-CoV-2, which is an enveloped, positive-sense single-stranded RNA virus, belonging to the beta coronavirus (β-CoV) 13, 14. The genome of SARS-CoV-2 comprises fourteen open reading frames (ORFs) 15, 16, that encode sixteen non-structural proteins (NSP), nine accessory proteins and four structural proteins. NSPs participated in the formation of replicase complex and remaining parts are involved in viral entry, assembly and release 15, 17. Spike protein (S) is critical for viral infection, which is an important target for combating SARS-CoV-2 18. S protein has a receptor-binding domain (RBD), an S1/S2 polybasic cleavage site and 3 O-linked glycans 19. All specific functional structures are the product of natural evolution.

**Figure 1:**
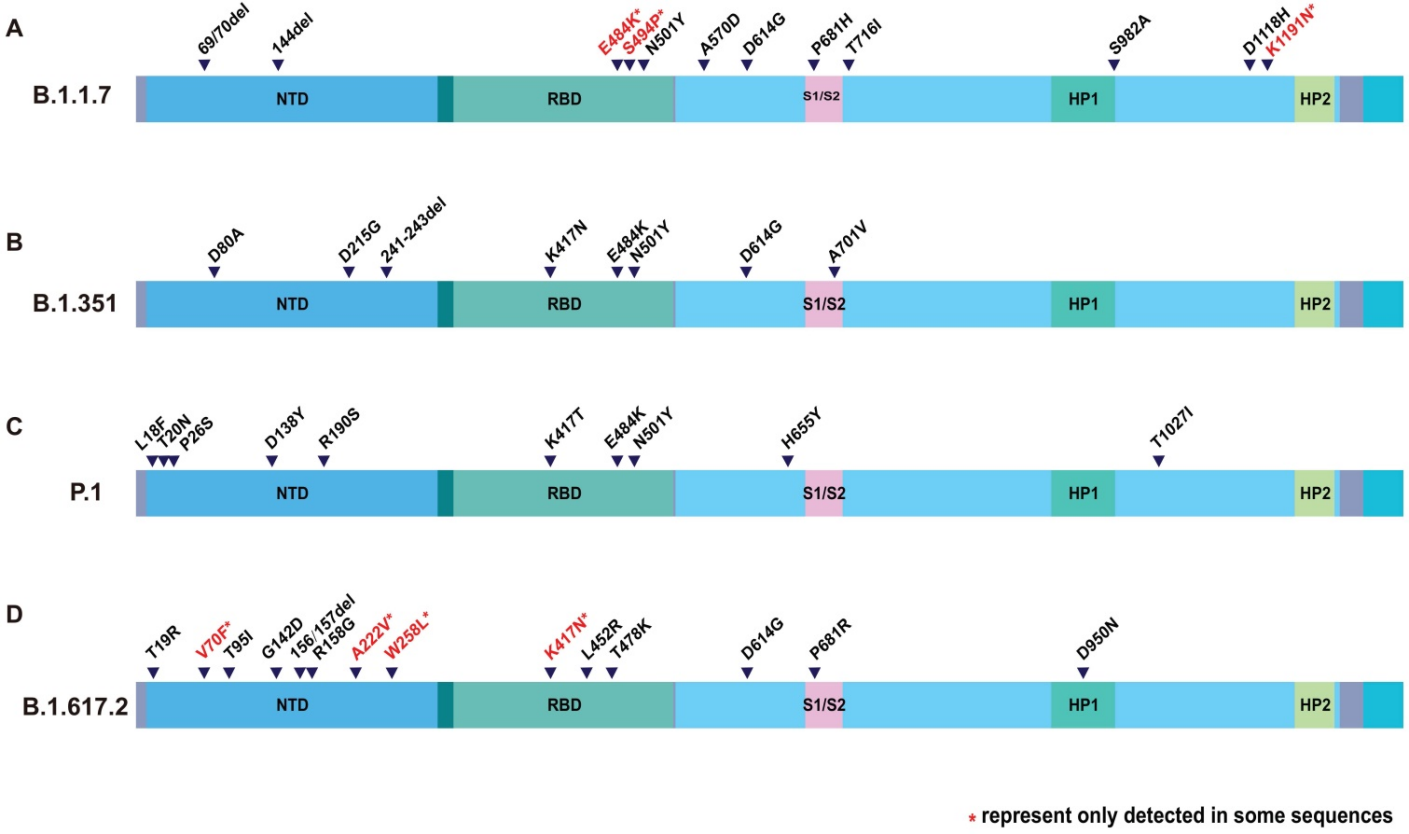
Amino acid changes in S protein of VOCs. Description of SARS-CoV-2 spike mutation in different virus strain. **(A)** B.1.1.7; **(B)** B.1.351; **(C)** P.1; **(D)** B.1.617.2. The colored columns describe the structural domain of spike protein. NTD: N-terminal domain; RBD: receptor-binding domain; HP1: heat protein 1; HP2: heat protein 2.

However, compared with the original virus, Delta is special. There are many mutations happened to Delta genome. Mutations in S protein are particularly abundant. According to the genome sequence analysis on GISAID, Delta lineage has 8 spike mutations (T19R, G142D, del157/158, L452R, T478K, D614G, P681R, and D950N) 20, 21. T19R, G142D, and del157/158 are located in the N-terminal domain (NTD), and L452R and T478K are in the receptor binding domain (RBD). Together with the D614G, all of the mutations mentioned above occur on the S1 subunit. P681R and D950N occur on the S2 subunit (Figure 1). It is interesting that N501Y was not found in Delta, but can be found in Alpha, Beta and Gamma variant. 22, 23 Neither does the E484K 24. Although Delta lacks these well-known mutations, that are common to multiple viral strains, it has produced many distinctive variants. For example, P681R, L452R and D950N. P681R resembled P681H of Alpha variant 25. L452R and D950N are new ones (Table 1). Distinguishing features make Delta a new viral strain.

### Clinical characteristics of Delta variant

The rapid spread of Delta virus is bringing a threat to us. Infection with Delta virus, causes flu-like symptoms 26. Fever, dry cough, weakness, coughing with sputum, headache, short breath, and aching pain in muscle were considered to be the common symptoms 27. Most patients had the first manifestation of hypoesthesia or loss of smell and taste 28. Some patients with severe disease often have dyspnea or hypoxemia one week after onset, others can quickly develop into acute respiratory distress syndrome (ARDS), septic shock, metabolic acidosis, coagulation dysfunction and multiple organ failure 29. Very few patients have the manifestations of central nervous system involvement and acral ischemic necrosis 30. There was no significant difference between the clinical characteristics of Delta virus infection and the symptoms caused by other strains.

## Transmission of Delta variant

According to the latest report issued by the WHO (Edition 51, published 3 August 2021), the cumulative number of patients with coronavirus infection reported globally is now nearly 197 million and the number of cumulative deaths is 4.2 million. Americas and Europe were the fastest growing regions in case incidence, and the Americas and South-East Asia Regions were reported to have the high mortality rate. Such scary number of infections is partly due to the prevalence of Delta virus.

### Global spread of Delta variant

Delta was first reported in India at the end of 2020 but have spread globally. By detecting throat and nose swabs from people who aged 5 years and older in England, Paul et al. found that B.1.1.7, which first appeared in the United Kingdom, was gradually replaced by Delta 31. And in the United States of America, since the first Delta infected person was detected in March, the dominant virus strain has also changed from B.1.1.7 to B.1.617 and P.1 32. Up to now, 135 countries have reported cases of the Delta variant 33, 34. Delta variant has strong transmission and increasing virulence.

### Possible reasons for rapid spread of Delta variant

**Figure 2:**
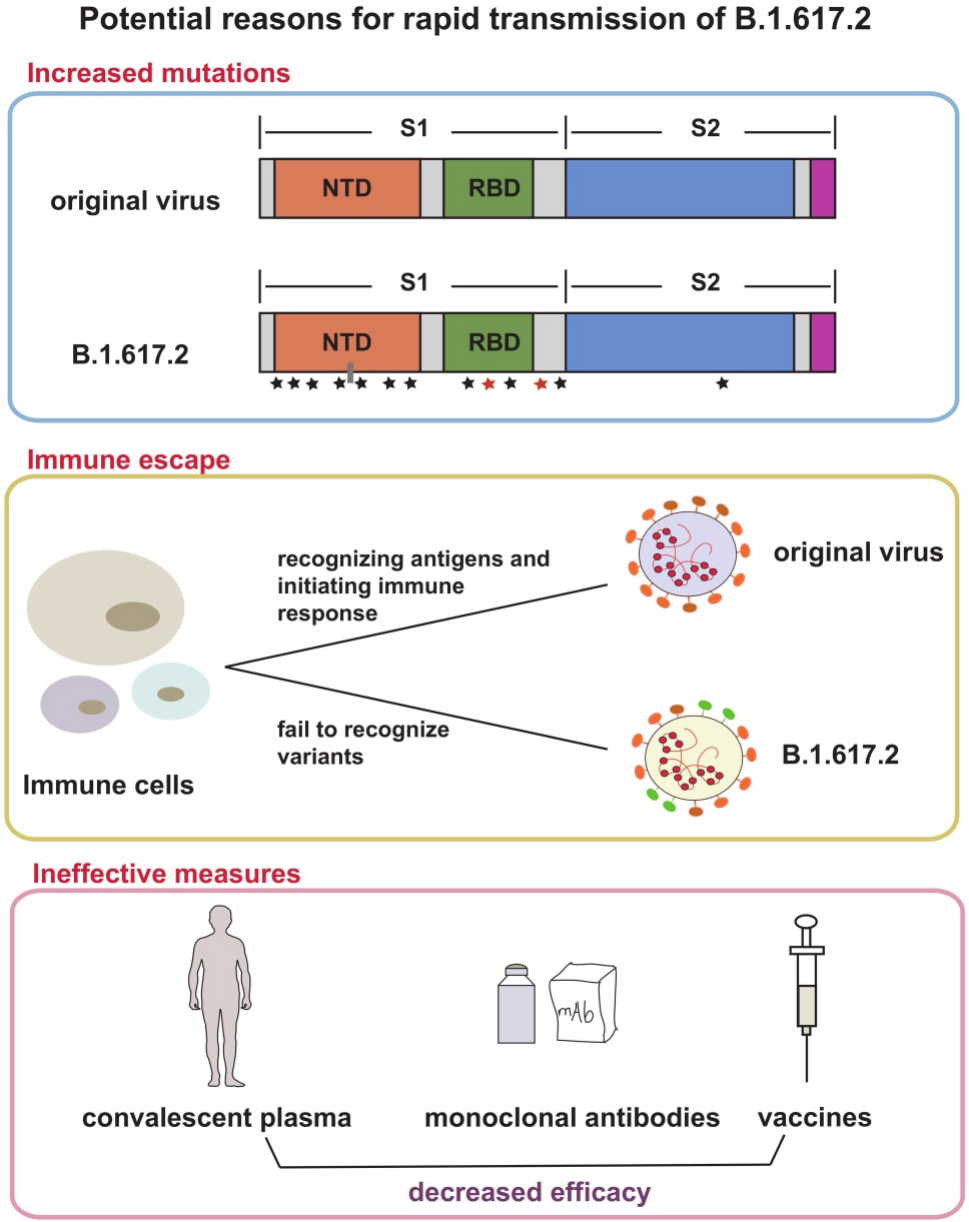
** Possible reasons for Delta variant rapid transmission.** This figure shows three reasons that explain the rapid spread of Delta variant and its emergence as the dominant mutant in many countries. The first frame suggested that the increased mutations in spike protein of Delta made contributions to transmission, the second frame depicts the occurrence of immune escape in Delta variant, and the third frame showed the decreased efficacy of convalescent plasma, monoclonal antibodies and vaccines. All above were potential reasons.

Based on current evidence, Delta variant is more transmissible than the Alpha variant. The ratio is about 40-60%, which may be related to greater risk of hospitalization. The factors driving the recent rapid growth of Delta-associated cases may be due to the followings (Figure 2): (1) More mutations and closer synergy. D614G exists in Delta genome. As the dominant viral strain in 2020, Delta is well known for its strong transmission and replication ability 35, 36. P681R changed the furin cleavage sites of the virus, which accelerated cell-cell fusion 37, 38. L452R increased the ability of virus to invade cells 39. Although the role of many other mutations has not been studied clearly, the diversity of mutations does make Delta more susceptible; (2) Immune escape. A recent study found that SARS-CoV-2 spike L452R variant, a critical variant in Delta, evades cellular immunity and increases infectivity 40 The same conclusion was also confirmed in another study 41; (3) Many protective measures are ineffective for Delta. A large number of experimental results suggested that current methods used for the prevention and treatment of infection do not work on Delta, including convalescent plasma 42, some monoclonal antibodies 43, and partial vaccines 43, 44; (4) Insufficient prevention and control. Shorten generation intervals or high transmissibility are responsible for Delta pandemic 45. Rigorous control could reduce these indicators.

## Potential strategies for combating Delta variant

Variations of SARS-CoV-2 is threatening, but there are still many ways to deal with it. Some direct and indirect strategies are listed below.

### Early diagnosis

Timely and effective diagnosis is necessary for the control of infectious diseases. Diagnosis of COVID-19 is the first step in the prevention and treatment. SARS-CoV-2 mutates frequently because of its unique genome structure and replication system. Reverse transcription loop-mediated isothermal amplification (RT-LAMP) is a powerful molecular tool for detecting SARS-CoV-2 RNA, including VOCs and VOIs. Alves et.al conducted a clinical validation of colorimetric RT-LAMP, they found that RT-LAMP is a fast and sensitive diagnostic tool and can be used to detect SARS-CoV-2 variants 46. Advances of CRISPR-Cas system indicates its significance in COVID-19 diagnosis, many excellent scientists build CRISPR-Cas-based system to identify infectious people. The most representative system are DETECTER 47 and SHERLOCK 48. CRISPR-Cas system provides us with fast and reliable detection methods 49. Early diagnosis is part and parcel of controlling Delta.

### Vaccines and monoclonal antibodies

Vaccines is a promising method to combating virus. Vaccination frequency is an important factor affecting the efficacy of mRNA vaccine. A study from NEJM shows that effectiveness after one dose of mRNA vaccine BNT162b2 was notably lower, only 30.7%. but the effectiveness of two doses has remarkable improvement, almost 88% 50. At the same time, an article published in the Lancet disclosed this result. This article also believes that mRNA vaccine can fight against COVID-19, but it is best to vaccinate with two doses 51. In addition to vaccination frequency, vaccination time and vaccination method will also have an impact on vaccine effectiveness. Zhang et al. found that vaccination in the morning produces a stronger protective immune response, which may be because the human immune system is affected by circadian rhythm 52. Hassan et al. show that ChAd-SARS-CoV-2-S, an adenovirus vector vaccine, can protect against SARS-CoV-2 invasion, and intranasally administered ChAd-SARS-CoV-2-S induces durable protection in BALB/c mice 53. The effectiveness of various vaccines on B.1.617.2 were summarized in Table 2. Monoclonal antibodies are equally important for curbing COVID-19. Although Bamlanivimab was proved ineffective, Etesivimab, Casirivimab and Imdevimab were proved to be useful 43.

### Structural biology assists in identifying drug targets

Before the advent of high-resolution crystal structures, it was common to combine biology, chemistry and physics to uncover the feature of proteins or small molecule compounds. However, the resolution is low and error prone, and the combination of the two substances cannot be revealed. Technological innovation has removed the obstacles for scientific research. Structural biology plays an essential role in antiviral research. structural biologists have used the advanced technology, x-ray crystallography (X-ray) and cryo-electron microscopy (cryo-EM), to analyze critical information. Yao et al. reported the molecular assembly of the authentic SARS-CoV-2 virus using cryoelectron tomography (cryo-ET) and subtomogram averaging (STA), which revealed SARS-CoV-2 panorama in a delicate manner 54, 55. Further refine the application of structural biology, receptor binding region 56, neutralizing antibody structure 57, spike mutation 58 are also within its scope. Using common viral strains, structural biology has helped people identify the key sites to break the virus and try to develop relevant inhibitors 59-61. Jin et.al found more than 10,000 potentially valid compounds through structure-based drug design and screening, and six of these compounds exhibited promising M-pro inhibited activity in cell-based assays 59. Beyond that, potential inhibitors are also identified by studying the complex structure of the drug binding to the virus 61-64. Although the research on antiviral drugs is still making efforts to be closer to clinical transformation, some non-antiviral drugs, such as Branebrutinib, have been successfully entered into clinical trials based on the help of structural biology 65. Therefore, we have reasons to trust structural biology to determine drug targets for Delta variant.

### Epidemic prevention and control

To protect ourselves from infected by Delta, we should not only rely on external measures, but also improve self-protection awareness. A large amount of evidence shows that the strength of government prevention and control, and the degree of citizens' compliance are positively correlated with the infection. Delta strain is special, but its biological characteristics have not undergone subversive changes. For this reason, wear masks, wash hands frequently, keep social distance and avoid crowded gathering are still worth advocating.

## Conclusions

After invading China, COVID-19 fights the world and defeats the defense lines of all countries, forcing WHO to declare that COVID-19 has reached the highest level of infectious disease. COVID-19 has spread worldwide over a year, and the extent and severity of the outbreak is deeply concerned. Many methods have been proposed to combat the virus, such as reduce exposure, drug treatment and vaccination, that can effectively delay the spread of the virus. While microorganisms are alive, they evolve in order to survive and reproduce, so is SARS-CoV-2. A lot of characteristic variations have been derived in the process of SARS-CoV-2 evolution. Delta is a SARS-CoV-2 variant originally found in India. It has spread to more than 135 countries in just half a year. At present, Delta has become the main variant in the world. The scientific community's understanding of Delta is still limited except its known infectious power. It is necessary to systematically expound the characteristics and global impact of Delta. In this article, we reviewed three major sections describing different aspects of Delta variant.

Firstly, we introduced features of Delta, including its biological features and clinical characteristics. Delta is a variant of SARS-CoV-2, similar to the original virus. But has its own distinguished feature. There are many remarkable mutations happened in S protein, for example, L452R and D950N. These mutations work together to make Delta virus become variant of concern.

Secondly, we described the dissemination of virus. Delta variant has become the leading variation and has involved in 135 countries. At the same time, a large number of studies show that the ultra-fast transmission speed not only increases the risk of infection, but also increases the hospitalization rate. Therefore, we summarized some reasons for this situation so that we can better understand and conquer Delta.

Finally, we proposed some possible measures for the treatment and prevention of Delta. We suggest that early diagnosis is important for control the infectious disease. Although there is no specific drug, vaccination is a reassuring treatment. There is a lot of evidence that the antibodies produced after vaccination can neutralize Delta variant. In addition, the latest research suggests that chimeric spike mRNA vaccines may be the direction to overcome the virus 66. Using structural biology to find potential drug target is also suggested. Last but not least, improving self-protection awareness never go out of style.

In conclusion, understanding the relevant information of Delta will help us fight it better. Only by knowing ourselves and others can we defeat the virus.

## Figures and Tables

**Table 1:** Characteristics of SARS-CoV-2 Variants of Concern

Variants of concern	Alpha	Beta	Gamma	Delta
Pango Lineage	B.1.1.7	B.1.351	P.1	B.1.617
First detected in	the United Kingdom	South Africa	Brazil	India
Earliest samples	September, 2020	May, 2020	November, 2020	October, 2020
Spike RBD mutations	E484K, S494P, N501Y	K417N, E484K, N501Y	K417T, E484K, N501Y	K417N, L452R, T478K
Spike non-RBD mutations	69/70del, 144del, A570D, D614G, P681H, T716I, S982A, D118H	D80A, D215G, 241/243del, D614G, A701V	L18F, T20N, P26S, D138Y, R190S, H655Y, T1027I	T19R, V70F, T95I, G142D, del157/158, A222V, W258L, D614G, P681R, D950N
Transmissibility	↑	↑	↑	↑
Virulence	↔	?	?	?

**Table 2:** Effectiveness of various vaccines on Delta variant

Name	Category	Country	Company	Effectiveness	Compared to other variants	Reference
BNT162b2	mRNA	USA	Pfizer	75%-82%	2.5-fold↓(compared with B.1.1.7 and P.1)	50, 67
ChAdOx1(AZD1222)	Viral vector	UK	AstraZeneca	53%-66%	4.3-fold↓(compared with B.1.1.7 and P.1)	67, 68
mRNA-1273	mRNA	USA	Moderna	76%	2.1 to 3.4 fold↓(compared with D614G)	69, 70
BBV152 /Covaxin	Inactivated	Indian	Bharat Biotech	65.2%	7-fold↓(compared with wildtype virus)	71
Sputnik V	Viral vector	Russia	Gamaleya Institute	69.85%	2.5-fold↓(compared with B.1.1.141 and B.1.1.317)	72
NVX-CoV2373	Recombinant SARS‐CoV‐2 Spike protein nanoparticle	USA	Novovax	NA	NA	73
CoronaVac	Inactivated	China	SinoVac	59%	NA	74
Ad26.COV2.S	Viral vector	USA	J&J/Janssen	60%	NA	75

## Abbreviations

SARS-CoV-2: Severe acute respiratory syndrome coronavirus 2
COVID-19: coronavirus disease 2019
VOC: variant of concern
WHO: World Health Organization
VOI: variant of interest
β-CoV: beta coronavirus
ORFs: open reading frames
NSP: non-structural protein
S: Spike protein
RBD: receptor-binding domain
NTD: N-terminal domain
ARDS: acute respiratory distress syndrome
RT-LAMP: reverse transcription loop-mediated isothermal amplification
CRISPR: clustered regularly interspaced short palindromic repeats
X-ray: X-ray crystallography
cryo-ET: cryoelectron tomography
STA: subtomogram averaging
